# Probability of Seeing Increases Saccadic Readiness

**DOI:** 10.1371/journal.pone.0049454

**Published:** 2012-11-14

**Authors:** Thérèse Collins

**Affiliations:** Laboratoire Psychologie de la Perception, Université Paris Descartes & CNRS, Paris, France; University of Muenster, Germany

## Abstract

Associating movement directions or endpoints with monetary rewards or costs influences movement parameters in humans, and associating movement directions or endpoints with food reward influences movement parameters in non-human primates. Rewarded movements are facilitated relative to non-rewarded movements. The present study examined to what extent successful foveation facilitated saccadic eye movement behavior, with the hypothesis that foveation may constitute an informational reward. Human adults performed saccades to peripheral targets that either remained visible after saccade completion or were extinguished, preventing visual feedback. Saccades to targets that were systematically extinguished were slower and easier to inhibit than saccades to targets that afforded successful foveation, and this effect was modulated by the probability of successful foveation. These results suggest that successful foveation facilitates behavior, and that obtaining the expected sensory consequences of a saccadic eye movement may serve as a reward for the oculomotor system.

## Introduction

In recent years, renewed interest on the importance of rewards for guiding behavior has emerged. Rewarding behavior can increase its frequency of occurrence, but can also exert an influence on more subtle levels of action preparation. For example, in a simple pointing task, the relative rewards and costs associated with different end positions influence the speed and precision of the movement [1]. In the oculomotor system as well, saccadic eye movements can reflect top-down variables such as the expected reward associated with a particular saccade, or the expected losses associated with erroneous saccades [2]. Milstein & Dorris [3], [4] showed that rewarding a saccade in a particular direction leads to increased saccadic readiness: rewarded saccades had shorter latencies and were harder to inhibit than non-rewarded saccades. Similar behavioral effects have been reported in primate studies, which allow investigation of the physiological correlates of the effects of reward on behavior [5]. For example, the basal ganglia neurons become more selectively tuned if the preferred direction is paired with a reward [6], [7]. These results suggest that saccades to rewarded directions are facilitated. In the monkey studies, reward was instantiated by a liquid reward, whereas in the human studies, reward was monetary: participants gained or lost a fixed amount of money as a function of their performance.

Therefore, while it is clear that the oculomotor system, like other sensory-motor systems, can express high-level cognitive processes such as the relative value or probability of reward, it is unclear what ecological value monetary rewards have. Money is a secondary reward, in contrast primary rewards such as food. The ecological value of such a primary reward is clear – to better move eyes and attention to probable food locations, whereas the reward value of secondary rewards is culturally dependent and learned.

The current study addresses the question of whether vision is a reward for the oculomotor system. The goal of saccades is to bring the high-acuity fovea onto areas or objects of interest, enabling detailed visual analysis. However saccades also have some costs, because perception is suppressed during saccades and mechanisms comparing pre- and post-saccadic images of the visual world occur to ensure a stable percept based on several retinal images that differ greatly [8]; saccade latencies may reflect these costs [9] The current study examined whether correctly acquiring the saccade target increased saccade readiness.

The basic idea was that successful foveation itself may serve as a kind of “reward” for the oculomotor system, such that movements that achieve foveation are facilitated relative to movements that do not. The reward studied here is therefore neither primary nor monetary, but informational: saccades usually lead to an increase in the amount of visual information available, and providing or withholding information may therefore influence saccade preparation. Movement facilitation is measured here by “saccadic readiness”, which corresponds to the speed with which a saccade can be made and the degree to which a reactive saccade to an abrupt visual onset can be suppressed. The general hypothesis was that saccades that were unlikely to acquire the visual target (i.e. to obtain the visual information that is usually the result of foveating) would become slower, less probable and easier to inhibit than saccades that were likely to acquire the visual target. The procedure was closely modeled on Milstein & Dorris [3], [4], and aimed to measure both the temporal and spatial aspects of saccade planning. Temporal planning was measured by examining saccade latency, the usual measure for assessing saccadic readiness. Fast saccades occur when the time needed to cross the threshold for saccade initiation is short, suggesting a baseline activity closer to that threshold. Spatial saccade planning was examined using the oculomotor capture task [10], [11]. Oculomotor captures occur when a saccade is directed to an irrelevant visual target appearing abruptly in the visual field. The capacity to inhibit the unwanted reactive saccade to this target is a measure of the baseline level of spatial saccadic preparation.

## Methods

The procedure was approved by the ethics committee of Paris Descartes University (Comité d'Evaluation Ethique en Recherche Biomédicale, CEERB).

### Subjects

Adult college-aged subjects (n = 22) performed saccades to targets that remained visible or disappeared upon saccade onset. Subjects participating in a single session volunteered (n = 14), subjects participating in three sessions (n = 8) received payment (10€/hour for approximately 2 hours). All provided informed consent.

### Stimuli and apparatus

Stimuli were presented on a 22″ Formac ProNitron 22800 screen with a resolution of 1024×768 and a refresh rate of 145 Hz. Fixation points were black 0.5°-diameter dots, distracters were 1°-diameter dots, and targets were 2°×4° photographs of human faces. There were 99 female and 169 male faces from the Raboud Faces Database [12] presented in black and white. Stimuli were presented on a gray background.

Movements of the right eye were monitored with an Eyelink 1 k (SR Research, Osgoode, Ontario, Canada) at 1000 Hz sampling rate. At the beginning of a session, the Eyelink was calibrated. Before each trial, fixation was checked. If the distance between the fixation check and the calibration was greater than 1.5°, fixation was refused and a new calibration was initiated. Calibration was also automatically renewed every 50 trials. On-line saccade detection was based on a boundary criterion: gaze-contingent target extinction occurred when the eye position crossed one half of the target eccentricity. Eye movement traces were subsequently analyzed offline. Instantaneous velocity and acceleration were computed for each data sample and compared to a threshold (30°/sec and 8000°/sec^2^). Saccade onset was defined as two consecutive above-threshold samples for both criteria. Saccade offset was defined as the beginning of the next 20-ms period of below-threshold samples.

### Behavioral tasks

**Figure 1:**
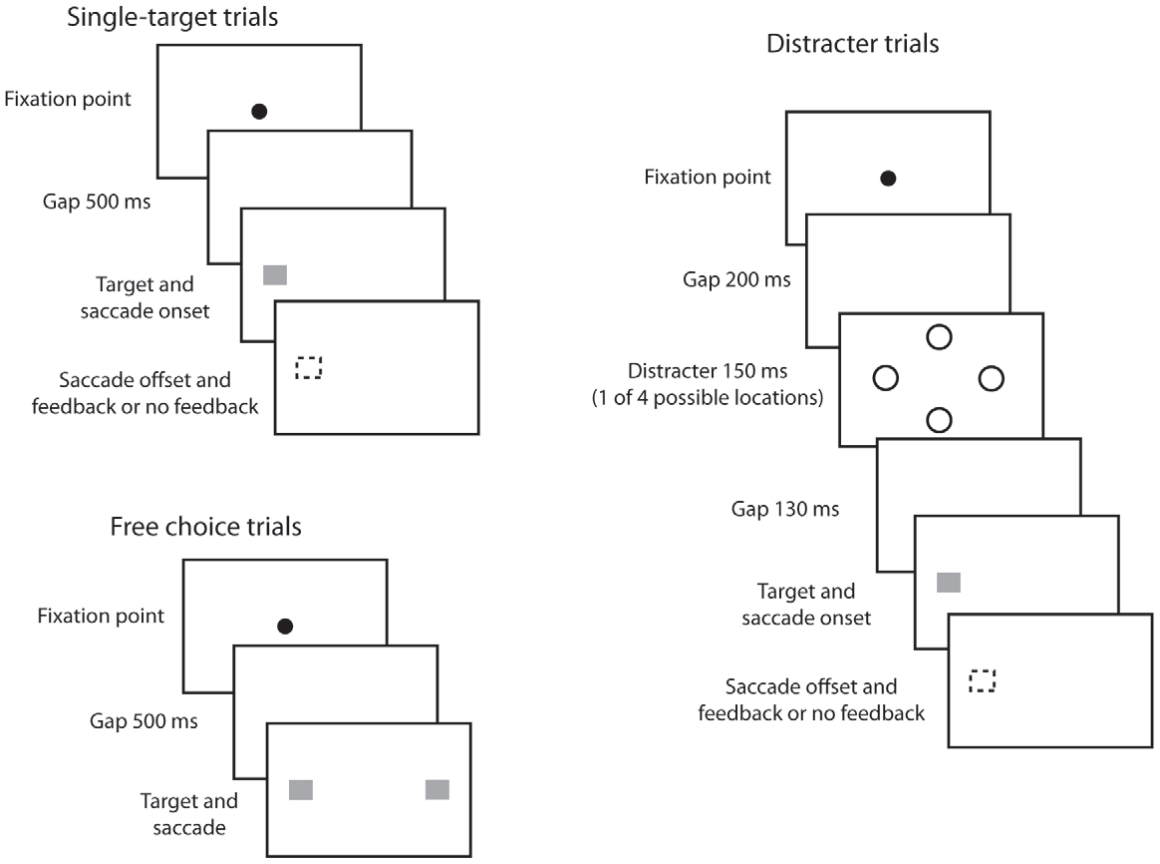
Procedure. Single-target and free choice trials: After successful fixation, a 500 ms gap was followed by presentation of one or two targets (single-target and free choice, respectively). Subjects had to saccade to (one of) the target(s). In the single-target trials, at saccade onset the target disappeared or remained visible depending on side and feedback condition. Distracter trials: After successful fixation, a 200 ms gap preceded the brief presentation of a distracter at one of four equiprobable locations. If an erroneous saccade to the distracter occurred, the trial was aborted. Otherwise, 130 ms after the distracter, a target appeared to the left or right and was extinguished or remained visible upon saccade onset, depending on side and feedback condition.

Subjects performed three tasks, intermixed but not equiprobable (Figure 1). They performed 24 practice trials before starting the first session. They were asked to report, at the end of the experiment, whether there were more female or male target faces. There was a majority (63%) of male faces and most subjects reported as such. The task was introduced to encourage subjects to actually foveate the targets.

#### Single-target trials

400–800 ms after successful calibration on the fixation dot (always at screen center), it was extinguished. 500 ms later, a target appeared 8° either to the left or to the right of fixation. Subjects were instructed to make a saccade to the target as fast as possible. Upon saccade detection, targets on the “no-feedback” side disappeared such that the eye landed on a blank screen while targets on the “feedback” side stayed on. All subjects performed a session in which feedback (no-feedback) targets remained visible (disappeared) on every trial (100% condition), while a subset (n = 8) performed two additional sessions in which feedback (no-feedback) targets remained visible (disappeared) in 75% or in 50% of trials. Feedback and no-feedback sides were counterbalanced across subjects, as was the order of the sessions in the subset of subjects who performed three. If subjects made a saccade before target appearance, a warning appeared (“fixate please”), the trial was aborted and put at the end of the trial cue. 120 of 204 total trials were single-target trials.

#### Free choice trials

500 ms after fixation dot extinction, two targets appeared 8° to the left and to the right of fixation. Subjects were instructed to make a saccade as fast as possible to the target of their choice. Targets did not disappear upon saccade detection, and subjects were able to inspect targets regardless of their side of presentation. If subjects made a saccade before target appearance, a warning appeared and the trial was aborted and put at the end of the trial cue. 20 of 204 trials were free choice trials.

#### Distracter trials

After successful fixation and a 200 ms gap, a distracter was flashed for 150 ms at one of four locations: 8° to the left, right, top or bottom. Subjects were instructed to ignore the distracter and continue fixating screen center. 130 ms later, a target appeared 8° to the left or right. Upon saccade detection, targets on the no-feedback side disappeared and targets on the feedback side remained visible (with the same probability as in the single-target trials). If subjects made a saccade to the distracter (“oculomotor capture”), the trial was aborted and the target was not presented. 64 of 204 trials were distracter trials.

### Data analysis

The 100% condition (n = 22) was analyzed with t-tests to compare the latency and choice behavior between feedback and no-feedback sides, and a one-way ANOVA to examine oculomotor captures according to distracter position (feedback, no-feedback, up, down). For the subset of subjects who performed all three feedback probability conditions (n = 8), latency and choice behavior were analyzed with two separate ANOVAs including side (feedback, no-feedback) and probability condition (100%, 75%, 50%) as factors, and oculomotor captures with an ANOVA with factors position and feedback probability. Note that for the 50% condition, there is no distinction between feedback and no-feedback sides, thus left versus right sides were compared in this condition. Huynh-Feldt corrections were made to the significance levels where appropriate (although uncorrected degrees of freedom are reported in the Results). Differences between feedback and no-feedback were subsequently analyzed with t-tests.

## Results

### Temporal saccade preparation

**Figure 2:**
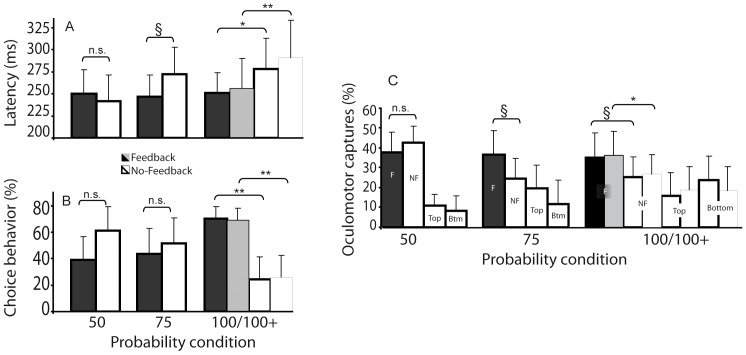
Saccadic performance averaged over all subjects in the 100% feedback probability condition (100+) and the subset of subjects who performed all three probability conditions (50%, 75%, 100%). The feedback side is in black (subset) or grey (all subjects), no-feedback side is in white with thick (subset) or thin (all subjects) outlines. Error bars represent SEM. § marginal (.051≤p≤.13, see text). * p<.05, ** p<.001. **A, B.** Latency and choice behavior (respectively) as a function of side (feedback; no-feedback) and feedback probability. **C.** Oculomotor captures as a function of side (F, feedback; NF, no-feedback; Top; Bottom) and feedback probability.

Temporal saccade preparation was assessed in the single-target trials (Figure 2A). In the 100% condition, saccade latency was 35 ms faster to the feedback side (256±45) than to the no-feedback side (291±55) (t(20) = 3.9, p<.001). In the subset of subjects who performed all probability sessions, latency depended both on side and on feedback probability (significant interaction, F(2,14) = 4.0, p<.045); Figure 1). Latencies were faster to the feedback side than to the no-feedback side, only in the 100% probability condition (difference of 26±24 ms; t(7) = 3.2, p<.016), and marginally in the 75% condition (difference of 12±38 ms; t(7) = 2.3, p<.051). Latencies in the 50% condition were similar for left and right sides (difference of −9±20 ms).

### Choice behavior

In the free choice trials (Figure 2B), subjects more often chose to saccade to the feedback side than to the no-feedback side, but only in the 100% probability condition (all participants: 73 versus 27%, difference of 45±35 percentage points; t(20) = 4.4, p<.001); subset: 74 versus 26%, difference of 48±19 percentage points; t(7) = 5.5, p<.001) (significant interaction between side and feedback probability, F(2,14) = 5.8, p<.015). In the 75% condition, choice behavior was roughly similar between feedback and no-feedback sides (46 versus 54% respectively, n.s.) and between left and right sides in the 50% probability condition (39% versus 61%, n.s.).

### Spatial saccade preparation

Spatial saccadic preparation was assessed in the distracter trials (Figure 2C). Oculomotor captures were more frequent to the feedback side than to the no-feedback side in the 100% probability condition (all subjects: 36 versus 27%, difference of 9±13 percentage points, t(19) = 2.4, p<.026; the degrees of freedom are different for this test because one of the subjects made no oculomotor captures in any condition). This difference did not hold up statistically in the subset of participants who performed all probability sessions, although the absolute size of the effect was similar (35 versus 25%, difference of 10±12 percentage points, t(6) = 1.8, p = .13). Oculomotor captures differed between feedback and no-feedback sides in the 75% probability condition (36 versus 25%) but the effect was marginal (t(6) = 2.1, p = .079), and not between left and right sides in the 50% probability condition (t<1).

Spatial saccade preparation may also be assessed by saccade endpoint accuracy. Indeed, in the single-target trials, there was a difference in saccade endpoints between feedback and no-feedback sides: saccades to the feedback side undershot slightly less than saccades to the no-feedback side (7.1±0.4° versus 7.0±0.9°; F(1,7) = 7.4, p = .03). Although descriptively, the effect was present in the 100% feedback condition (7.4° versus 6.9° for feedback and no-feedback sides respectively) but less so or not at all in the 75% (6.9±1.1° versus 7.0±0.6°) and 50% (7.1±0.8° versus 7.0±0.5°), there was no statistically significant effect of probability condition (F<1) and no interaction between the two factors (F<1).

There was a correlation between temporal and spatial saccade preparation: the greater the latency difference between feedback and no-feedback sides, the greater the capture difference as well. All individual slopes relating the latency difference to the capture difference were positive, and the average slope was thus greater than 0 (one-tailed t-test, p<.02); the overall R^2^ was 46%.

## Discussion

Throughout the course of the experiment, subjects learned that targets at one location were more likely to remain visible after the saccade than targets at other locations. The probability of seeing a target enhanced the level of saccadic readiness, as assessed by latency, choice behavior and oculomotor capture. Specifically, saccades to those targets were faster, more frequent, and harder to inhibit than saccades to other targets. The increase in saccadic readiness parallels results found in studies using monetary reward in humans [2], [3]. Post-saccadic visual feedback may thus act as a reward for the visuo-motor system, such that saccades that are likely to achieve successful foveation and acquire visual information are facilitated relative to saccades that are not. The nature of the reward afforded by foveation can be described in terms of informational content: saccades that lead to an increase in visual information are facilitated. This parallels studies showing that latency is faster when a saccade is necessary to perform an attentional task relative to saccades in a task that can be performed during the current fixation [9]. In the present study, there was no task in each trial; observers had to identify targets for a global report at the end of the experiment.

In the Milstein & Dorris [2] study, the probability of reward (percentage of trials on which a reward was delivered) and the magnitude of that reward (amount gained) were differentiated. The best predictor of saccadic readiness was a combination of probability and magnitude of reward. In the present study, the magnitude of reward was not varied: the visual target was either present or absent on any given trial. In further studies, it may be possible to vary the magnitude of “visual reward” to examine whether, as with monetary reward, both probability and magnitude of reward influence saccadic readiness.

The current results may be related to the finding that the probability of appearance of a visual target reduces the latency of saccades directed to it [13]. According to the LATER model, saccadic latency depends on the build-up of activity in saccade centers (e.g. [14]), execution occurring when the activity crosses a certain threshold [15]. The rate of rise depends on the evidence in favor of target presence, and the start level depends on the probability of the stimulus appearing: saccades to more frequent targets are faster because the level of activity is increased for these targets specifically [13]. Target probability increases saccadic readiness rather than increasing the responsiveness of saccadic centers to visual information (rate of rise). In the current study, targets to the left and right sides were equally probable, but the frequency with which they remained available for post-saccadic visual inspection was not. Although insufficient data was collected to run a satisfactory simulation of the data according to the LATER model, it is tempting to speculate that increased probability of seeing also elevated the starting level of activity.

The current results are compatible with several previous studies in monkeys that revealed the neuronal mechanisms that might sub-tend the behavioral effects [5]. For example, caudate nucleus neurons develop a spatial bias for rewarded positions. The bias appears when the monkey is waiting for the go-signal to make a saccade, suggesting that activity to the rewarded side is elevated before target onset [7]. Such an elevated baseline activity in topographically organized saccade maps could cause the readiness effects seen behaviorally; a candidate structure may be the superior colliculus [16], [17]. When saccades in different directions give rise to different rewards, the baseline activity of neurons at a particular location on these maps may be elevated relative to others, particularly those controlling saccades to non-rewarded directions.

In humans, selectively rewarding some saccades also influences saccade amplitude and variability [18], [19]. In these studies, saccades of a particular amplitude were “rewarded” by a tone presented at saccade offset, while “non-rewarded” saccades received no auditory feedback. The mean amplitude of subsequent saccades shifted to match the average amplitude of saccades that had received auditory feedback. Although it remains unclear why a tone should represent a reward for the visuo-motor system, these studies suggest that movement parameters can be modified following the modification of the sensory consequences of those movements.

In this way, the present results may shed light onto the mechanisms recent studies in neuroeconomics have uncovered. The sensitivity of saccades, and body movements in general, to the value associated with different objects is reflected in movement parameters. Behavioral effects of monetary reward may reflect high-level cognitive control over movement preparation. It may also be the case that sensory-motor systems have their own intrinsic reward, such as successful foveation for the visuo-ocular system. Foveating a target allows the visual system to process information in a manner not possible when the target is peripheral. The increase in informational content as a result of saccades is probably a more relevant “currency” for the oculomotor system than monetary or primary reward. The informational content may simply be a clearer view of visual objects, allowing fine-grained analysis. It may also be the case that obtaining sensory consequences that match the predicted consequences of movement, based on a forward model [20], serves to reinforce behavior. In this view, the relevant informational consequence of a saccade is not restricted to immediate visual processing, but also plays a role in ongoing movement monitoring in the service of learning.
